# ‘*Candidatus* Cochliophilus cryoturris’ (Coxiellaceae), a symbiont of the testate amoeba *Cochliopodium minus*

**DOI:** 10.1038/s41598-017-03642-8

**Published:** 2017-06-13

**Authors:** Han-Fei Tsao, Ute Scheikl, Jean-Marie Volland, Martina Köhsler, Monika Bright, Julia Walochnik, Matthias Horn

**Affiliations:** 1 Division of Microbial Ecology, Department of Microbiology and Ecosystem Science, University of Vienna, Vienna, Austria; 2 Institute of Specific Prophylaxis and Tropical Medicine, Medical University of Vienna, Vienna, Austria; 3 Department of Limnology and Oceanography, University of Vienna, Vienna, Austria

## Abstract

Free-living amoebae are well known for their role in controlling microbial community composition through grazing, but some groups, namely *Acanthamoeba* species, also frequently serve as hosts for bacterial symbionts. Here we report the first identification of a bacterial symbiont in the testate amoeba *Cochliopodium*. The amoeba was isolated from a cooling tower water sample and identified as *C. minus*. Fluorescence *in situ* hybridization and transmission electron microscopy revealed intracellular symbionts located in vacuoles. 16S rRNA-based phylogenetic analysis identified the endosymbiont as member of a monophyletic group within the family Coxiellaceae (Gammaprotebacteria; Legionellales), only moderately related to known amoeba symbionts. We propose to tentatively classify these bacteria as ‘*Candidatus* Cochliophilus cryoturris’. Our findings add both, a novel group of amoeba and a novel group of symbionts, to the growing list of bacteria-amoeba relationships.

## Introduction

Free-living amoebae are ubiquitous unicellular eukaryotes found in a wide range of habitats ranging from soil and aquatic environments to dust and air^1, 2^. Grazing upon other microbes, they are important predators shaping microbial communities and affecting ecosystem functioning including nutrient availability and mineralization^3^.

Free-living amoebae are also known as hosts for diverse bacteria and giant DNA viruses^4–6^. They serve as reservoirs for a number of human pathogens such as *Legionella pneumophila* ^7^, *Pseudomonas aeruginosa* ^8^, *Francisella tularensis* ^9^, *Coxiella burnetii* ^10^ *, Vibrio cholerae* ^11, 12^, *Aeromonas hydrophila* ^13^, and *Mycobacterium* species^14^, all of which escape the regular phagolysosomal pathway and transiently replicate within amoeba trophozoites. In addition, long-term stable associations between obligate intracellular bacteria and amoebae have been reported, with known symbionts being affiliated with the bacterial phyla Chlamydiae, Alphaproteobacteria, Betaproteobacteria, Gammaproteobacteria, Bacteroidetes, and the candidate phylum Dependentiae (formerly TM6)^5, 15–19^.

Bacteria-amoeba relationships can have diverse effects on both partners. Enhancement of bacterial virulence upon amoeba passage has been reported as well as an increased cytopathogenicity of the amoeba host cell in the presence of bacterial symbionts^20–22^. Amoeba-associated bacteria share a number of characteristic genomic features; amoebae have thus been proposed to represent “melting pots” facilitating horizontal gene transfer between bacterial symbionts^23–26^. In addition, amoebae may have served as evolutionary training grounds for bacterial pathogens by providing conditions favoring bacteria with enhanced pathogenicity^27–29^.

Bacteria-amoeba relationships have been studied almost exclusively in *Acanthamoeba* and few *Vanella* and *Vermamoeba* (formerly *Hartmannella*) isolates, all of them being free-living naked amoebae. Here we analyzed an amoeba newly recovered from a cooling tower water sample and identified as belonging to the testate amoebae *Cochliopodium*. These amoebae are covered with a tectum, a dorsal scale-like carbohydrate cell coat that protects the plasma membrane^30^. About 20 species have been recognized; they are primarily found in freshwater, brackish-water and marine environments, and rarely in soil^31–35^. The genus *Cochliopodium* represents a monophyletic group within the Amoebozoa, order Himatismenida, which forms a sister clade of the Centramoebida (containing the genus *Acanthamoeba* and others)^36^.

Here we report on the first characterization of a bacterial symbiont found in the testate amoeba *Cochliopodium*. The rod-shaped, Gram-negative bacterial symbiont replicates in host-derived vacuoles within its amoeba host cell and represents a distinct, yet uncharacterized lineage within the family Coxiellaceae (Gammaproteobacteria, Legionellales).

## Results and Discussion

### *Cochliopodium minus* from a cooling tower water sample

**Figure 1:**
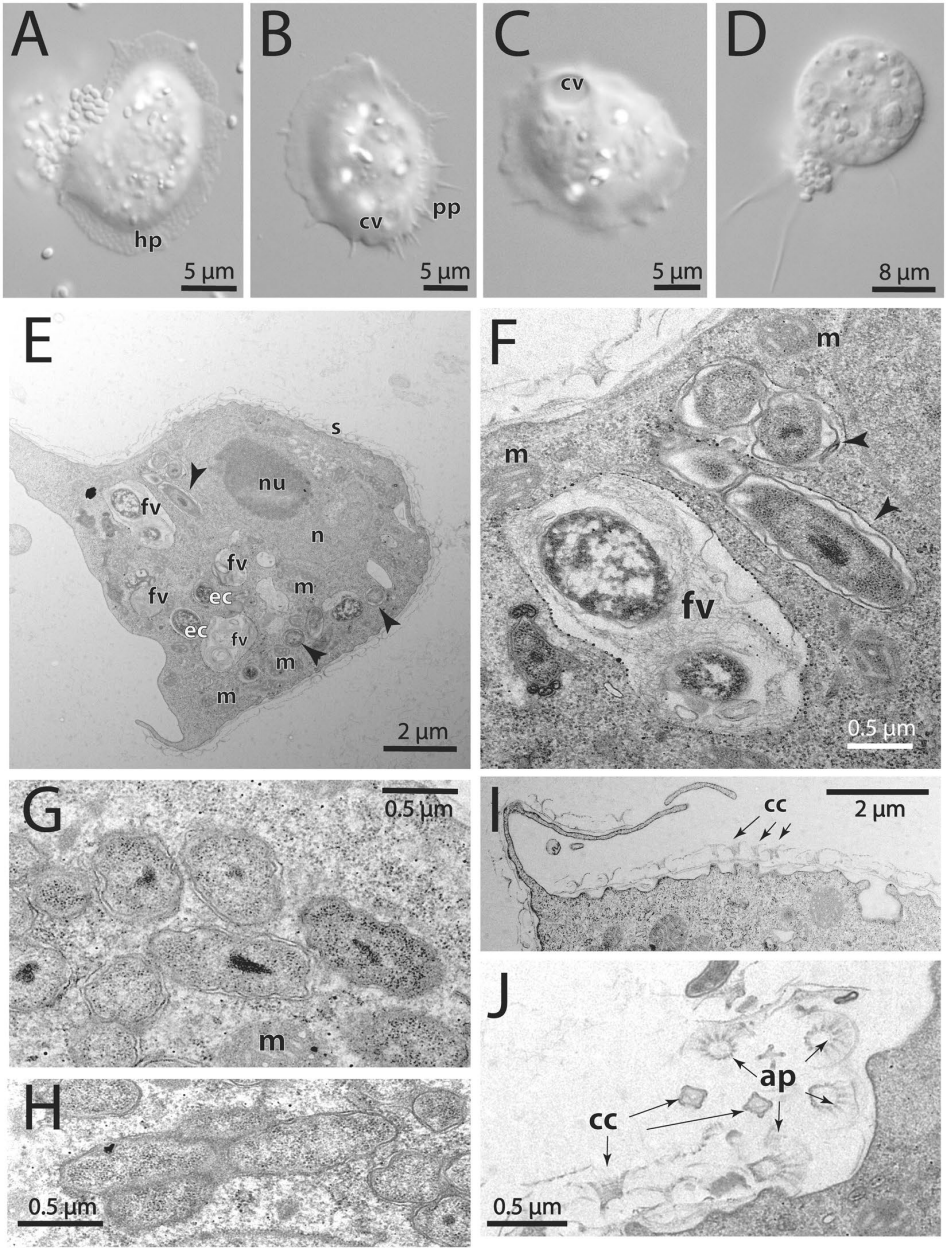
Intracellular location and morphology of ‘*Candidatus* Cochliophilus cryoturris’ PDD8 in *Cochliopodium minus* 9B. (**A**–**C**) Lightmicrographs of *Cochliopodium minus* trophozoites with the characteristic scaled hyaloplasm (hp); the contractile vacuole (cv) involved in osmoregulation and subpseudopodia (pp) are readily visible. (**D**) Early stage of the encystation process showing the onset of cytoplasm condensation. (**E**) Electronmicrograph of a trophozoite containing several *C. cryoturris* symbionts (arrow heads) and food vacuoles (fv) with *E. coli* (ec; note the evidence for degradation). The host nucleus (n), the nucleolus (nu), and the dorsal scale cover (s) can be recognized. (**F**–**H**) *C. cryoturris* is located in membrane-bound compartments (arrow heads); the bacteria show a Gram-negative type cell envelope, with a partially widened periplasmic space; an electron-dense central area indicating condensed cytoplasmic components is present in many of the symbionts; mitochondria (m) can be seen in the vicinity of symbiont-containing vacuoles. (**I**) The characteristic scales of the *Cochliopodium minus* host are shown in a cross section including the funnel-shaped central column (cc). (**J**) The apical part (ap) of the scales as well as vertical and tangential sections of central columns (cc) are visible.

During a cooling tower water screening study^37^, an amoeba was isolated that could be readily propagated on non-nutrient agar plates coated with *E. coli*. Our attempts to establish an axenic culture using various media and a hypersensitive *E. coli* mutant^38^ failed, and the amoeba was thus maintained routinely on agar plates. Morphological analyses, along with molecular identification based on 18S rRNA gene sequencing, confirmed the classification of this isolate as *Cochliopodium minus*, a testate amoeba found in diverse marine and fresh-water environments^31–35^. Highest 18S rRNA sequence similarity (>99%) was observed with *Cochliopodium* sp. F-117 (ATCC® 30936™) and various *Cochliopodium minus* strains. Characteristic for members of the genus, the trophozoites of this new isolate are covered with a dorsal monolayer of scale-like structures, the so called tectum (Fig. 1A,E,I,J). Lightmicrographs show the thin, scaled-covered hyaloplasmic sheet surrounding the granuloplasm, as well as the presence of subpseudopodiae (Fig. 1A,B). Contractile vacuoles of various stages, able to undergo fusion, are present in the cytoplasm (Fig. 1B,C). Occasionally, we could observe various encystation stages, including rounded trophozoites, the beginning of the encystation process (Fig. 1D). Transmission electron microscopy demonstrated that arrangement and fine structure of the scales are consistent with those described for other *Cochliopodium minus* isolates^39^ (Fig. 1I,J). We thus refer to the novel isolate as *C. minus* strain 9B. It is interesting to note that another *C. minus* isolate was previously reported to contain bacterial symbionts, which could not be further characterized at the time^40^.

**Figure 2:**
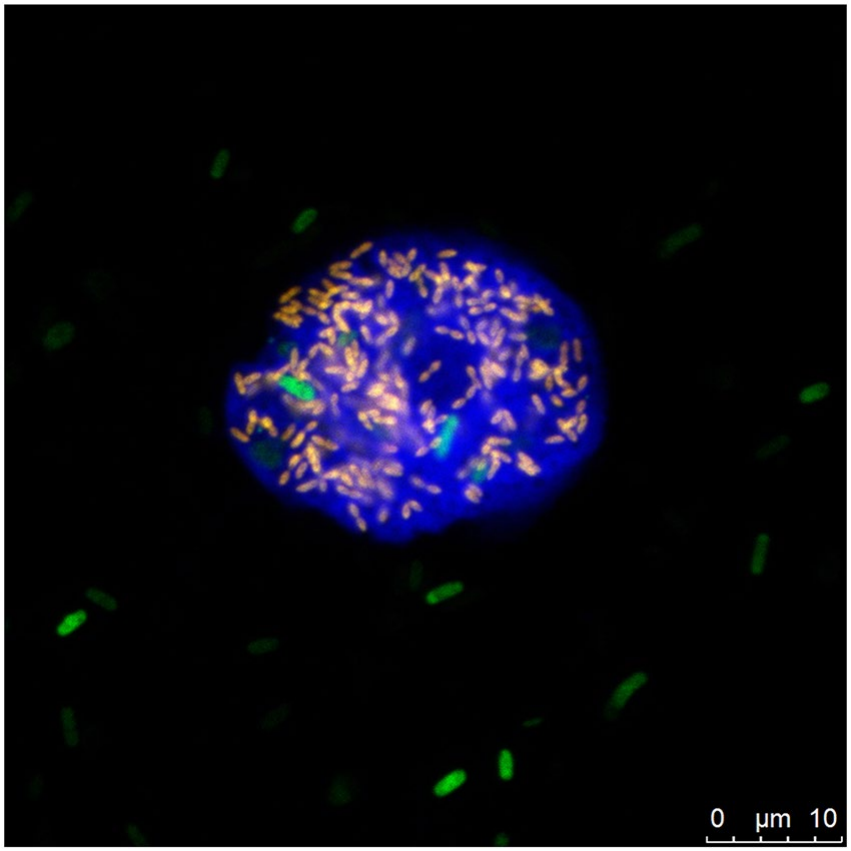
Identification of ‘*Candidatus* Cochliophilus cryoturris’ PDD8 in its native host *Cochliopodium minus* by fluorescence *in situ* hybridization. The bacterial endosymbionts were visualized with the specific probe PDD8-644 labeled with Cy3 (red) and general bacterial probes (EUB-mix) labeled with Fluos (green; the overlap appears yellow). *E. coli* cells added as amoeba food source are visible in green; the *Cochliopodium minus* trophozoite was counterstained using the eukaryotic probe EUK516 labeled with Cy5 (blue).

Intracellular bacteria in *Cochliopodium minus* 9B Staining of *C. minus* 9B trophozoites with the DNA dye DAPI readily revealed small, rod-shaped bacteria within the amoeba cytoplasm that differed in fluorescence intensity, quantity and size from *E. coli* cells, which were primarily observed outside of the trophozoites (data not shown). The presence of bacteria other than *E. coli* was further confirmed by fluorescence *in situ* hybridization (FISH), and 16S rRNA gene sequencing recovered a sequence with highest similarity to members of the bacterial order Legionellales (Gammaproteobacteria). We designed an oligonucleotide probe for the specific detection of this sequence; its application in FISH together with general bacterial and eukaryotic probes demonstrated unambiguously the presence of bacterial endosymbionts in *C. minus* (Fig. 2). All analyzed trophozoites were infected, and the bacteria were always located in the amoeba cytoplasm, being notably absent in the nucleus^19, 41^. The number of symbionts per amoeba cell varied and ranged from a few to over 100. The infection did not compromise the host’s capability to encyst as described for some other symbionts^16, 42^, nor did we observe pronounced lysis of the amoeba at room temperature or at 28 °C. Over a period of two years, the amoebae remained infected, demonstrating that this symbiont-amoeba relationship is a stable long-term association. Failed attempts at host-free cultivation in diverse nutrient-rich and complex media under oxic and micro-oxic conditions indicate that the symbiont is dependent on its amoeba host and should therefore be considered obligate intracellular.

### A novel clade of endosymbionts in the Coxiellaceae

**Figure 3:**
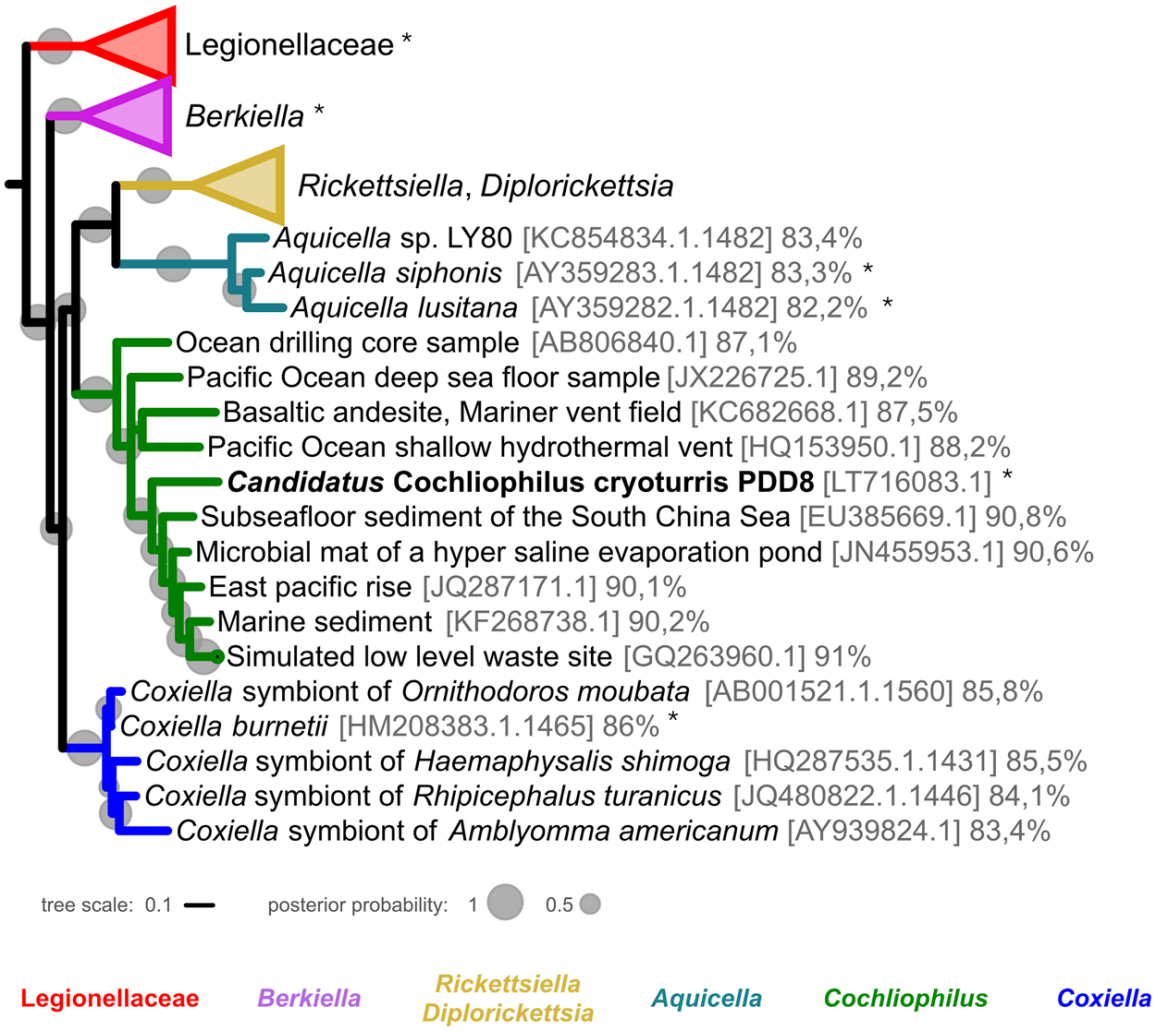
Relationship of ‘*Candidatus* Cochliophilus cryoturris’ PDD8 with other members of the Legionellales. A 16S rRNA tree based on PhyloBayes using the CAT model and GTR exchange rates is shown. Posterior probability values are indicated as grey circles at the nodes. Accession numbers and sequence similarity values to *C. cryoturris* are provided. Taxa with reported amoeba association are labeled with an asterisk. *C. cryoturris* together with a number of sequences obtained from various aquatic samples forms a novel sister clade of the *Rickettsiella/Diplorickettsiella/Aquicella* group in the family Coxiellaceae.

The near full length 16S rRNA sequence (1,506 bp) of the *C. minus* 9B symbiont showed highest sequence similarity to a clone sequence from a soil sample (91%; accession number GQ263960.1). The most similar cultivated representative was *Coxiella burnetii* RSA 331 (CP000890.1), with only moderate sequence similarity (86%). Phylogenetic analysis confirmed that the symbiont is affiliated with the order Legionellales, in which it forms a well-supported monophyletic group together with a number of uncultured microbes predominantly from diverse marine environments (Fig. 3). This yet uncharacterized group represents a sister clade of the *Rickettsiella/Diplorickettsiella/Aquicella* group, three genera in the family Coxiellaceae. Notably, the bacterial symbiont of *C. minus* 9B is not closely affiliated with any known amoeba endosymbiont. However, its moderate relationship with members of the Legionellales is intriguing, as this order comprises a number of bacterial taxa associated with eukaryotes, including human and animal pathogens as well as parasites of amoebae (Fig. 3).

Members of the genera *Rickettsiella* and *Diplorickettsia* are parasites and symbionts of arthropods, including insects, crustaceans, and arachnids^43^. *Aquicella* species were first isolated from borehole and spa water samples and later shown to be able to thrive in co-culture with *Vermamoeba vermiformis* ^44^. The genus *Coxiella* currently includes a single recognized species, *C. burnetii*, with numerous pathovars; these obligate intracellular bacteria are associated with insects and can cause severe infections in humans (aka Q fever)^45^; they might also be able to thrive in *Acanthamoeba castellanii* ^10^. *Berkiella* species have recently been identified as intranuclear symbionts of *Acanthamoeba polyphaga* ^41^. Furthermore, the Legionellaceae comprise a large number of facultative intracellular bacteria able to infect protists and animals including humans^46, 47^.

Taking into account current thresholds for the delineation of bacterial genera and families^48^, the *C. minus* symbiont identified in this study represents a novel genus. We thus propose to tentatively classify this microbe as *Cochliophilus cryoturris* PDD8 (Cochliophilus, pertaining to the obligate intracellular lifestyle in its native host *Cochliopodium minus*; cryoturris, pertaining to the origin of the water sample, a cooling tower, from which the amoeba host was isolated). Currently, the novel genus is placed within the Coxiellaceae, although we noted that the sequence similarity of *C. cryoturris* and its relatives to other members of the Coxiellaceae is below the commonly used family level threshold of 86.5% (Fig. 3)^48^. *C. cryoturris* is currently the sole isolated representative of this novel genus; whether the other uncultured members of this clade are also naturally associated with protists is still unclear.

### Vacuolar location of *C. cryoturris*

The appearance and host-associated intracellular lifestyle of *Cochliophilus cryoturris* PDD8 is reminiscent of that of many of its relatives in the Legionellales. *C. cryoturris* cells are small and show a short rod-shaped morphology, measuring 0.5 ± 0.1 µm in width and around 1 ± 0.2 µm in length; they show a Gram-negative type cell envelope and frequently a condensed, electron-dense central region in the cytoplasm (Fig. 1F,G). The symbionts are not located directly within the amoeba cytoplasm but in membrane-bound compartments (Fig. 1E,F). These clearly differ from the food vacuoles containing (degraded) *E. coli* cells, which can also be seen in the amoeba cytoplasm (Fig. 1E,F). Mitochondria are frequently located in the vicinity of the symbiont-containing vacuoles (Fig. 1E–H).

Bacterial strategies for escaping phagolysosomal degradation differ. *Coxiella burnetii*, the closest cultured relative of *C. cryoturris*, is able to resist the harsh conditions after fusion of the phagosome with lysosomes and modifies the phagolysosome to interact with the autophagic pathway, promoting metabolic activity and replication^49, 50^. *Legionella pneumophila* takes an alternative route and prevents lysosomal fusion to establish a heavily modified vacuolar compartment resembling endoplasmic reticulum (ER) membranes^51, 52^. How *C. cryoturris* establishes its intracellular niche is currently unknown. We were, however, unable to infect different *Acanthamoeba* species, which are otherwise permissive to an array of phylogenetically diverse intracellular bacteria. This suggests that *C. cryoturris* is well adapted to infection of its natural *Cochliopodium* host but might have a limited host range.

In conclusion, we discovered and identified the first naturally occurring *Cochliopodium* endosymbiont together with its amoeba host. The symbiont is a representative of a hitherto uncharacterized clade of microbes found in diverse aquatic environments and related to other intracellular bacteria in the family Coxiellaceae. Together this indicates that relationships between free-living amoebae and bacterial symbionts are more widespread than currently recognized.

### Description of ‘*Candidatus* Cochliophilus cryoturris’ PDD8

*Cochliophilus cryoturris (*Cochliophilus, pertaining to the obligate intracellular lifestyle of the bacteria in the native host *Cochliopodium minus;* cryoturris, pertaining the origin of the water sample from which the amoeba host was recovered, a cooling tower). Phylogenetic position: *Proteobacteria*; *Gammaproteobacteria*; *Legionellales*; *Coxiellaceae*. Rod-shaped Gram-negative bacteria; 0.5 µm in width and 1 µm in length. Obligate intracellular symbiont of *Cochliopodium minus* 9B; residing in membrane-bound compartments. The amoeba host was isolated from water samples of a cooling tower in Vienna, Austria (18S rRNA gene sequence accession number at Genbank/ENA/DDBJ KU215597). Basis of assignment 16S rRNA gene (Genbank/ENA/DDBJ accession number LT716083) and oligonucleotide probe PDD8-644 (5′-TCTTCGACTCCAGCCGCAC-3′; http://probebase.net/pb_report/probe/4042).

## Material and Methods

### Amoeba isolation and cultivation

A water sample was collected from the tank-bottom of a cooling tower and stored at 4 °C. 250 µl were filtered onto a cellulose-nitrate filter (Sartorius Lab Instruments GmbH & Co. KG, Göttingen, Germany; pore-size 0.2 µm). The filter was cut into two pieces, which were transferred onto non-nutrient agar plates (PAS; 0.12 g l^−1^ NaCl, 0.004 g l^−1^ MgSO_4_ *7 H_2_O, 0.004 g l^−1^ CaCl_2_*2H_2_O, 0.142 g l^−1^ Na_2_ HPO_4_, 0.136 g l^−1^ KH_2_PO_4_, 1.5 g l^−1^ agar) coated with *Escherichia coli* and stored at room temperature. Detected amoebae were cloned by daily serial sub-culturing of single cells onto fresh *E. coli*-coated agar plates using a sterile inoculation loop. Clonal cultures were maintained by weekly sub-culturing and morphological identification of the amoeba was accomplished by inverted phase contrast and bright field microscopy (Nikon Eclipse E800) using the identification keys of Page and Smirnov^53, 54^. Amoebae thriving on the agar surface were repeatedly transferred to fresh agar plates containing *E. coli* JW5503-1 ΔtolC732::kan as food source to facilitate axenization as described^38, 55^. Amoeba were routinely maintained on agar plates covered with *E. coli*. Fresh bacteria suspended in PAS were added to the agar plate once per week, and once per month an agar piece was transferred to a new plate.

To facilitate microscopic and molecular analysis, an agar piece containing amoebae was transferred to a culture flask (Nunclon delta-surface, Thermo Scientific, St. Leon-Rot, Germany) containing 6 ml PAS. Amoebae were allowed to attach for 12 h and washed twice with PAS to remove *E. coli*. Amoeba cells were collected for further analysis by detaching through vigorous shaking.

### Infection of *Acanthamoeba*

Amoebae were harvested from cell culture flasks, and the cell suspension was transferred to a Dounce tissue grinder (Sigma-Aldrich Handels GmbH, Vienna, Austria) using the tight pestle for 15 times to break up the cells and release the endosymbionts. The suspension was filtered (Macherey-Nagel, Düren, Germany; pore size 5 µm) twice to remove remaining intact amoebae. The symbiont suspension was added to cultures of *Acanthamoeba castellanii* Neff (ATCC 30010)*, Acanthamoeba* sp. 5a2^18^, and *Acanthamoeba* sp. UWC12^56^. The outcome of the infection progress was monitored by fluorescence *in situ* hybridization.

### Host-free cultivation attempts

Host free growth of purified symbionts was tested using the following media: peptone-yeast-glucose (PYG) broth, trypticase-soy broth with yeast extract (TSY), peptone-yeast-nucleic acids-folic acids-hemin (PYNFH) broth, buffered charcoal yeast extract (BCYE) medium with and without supplements. Micro-oxic conditions were applied using the CampyGen Compact system (Thermo Scientific, St. Leon-Rot, Germany).

### Transmission electron microscopy

Amoebae were fixed (2.5% glutaraldehyde in 3 mM cacodylate buffer containing 0.1 M sucrose, pH 6.5) in culture flasks for one hour, detached using a cell-scraper, and concentrated by centrifugation (2900 rcf, 6 min). The pelleted cells were washed with 0.1 M cacodylate-sucrose buffer (pH 7.2–7.4) for three times and then resuspended in 40 µl 1% agarose (Low melting point agarose; Promega, Mannheim, Deutschland). The agar pellet was solidified on ice for 45 min and then cut into smaller pieces with 1 mm thickness, which were fixed in 1% OsO_4_ for 1 h and dehydrated in an increasing ethanol series. Agar blocks were embedded in Low Viscosity resin (Agar Scientific®) and polymerized for 48 h at 60 °C. Ultrathin sections placed on Formvar® coated slot grids were stained with 0.5% uranyl acetate and 3% lead citrate prior to imaging with a Zeiss® Libra 120 transmission electron microscope.

### Fluorescence *in situ* hybridization

An agar piece containing amoebae was placed upside-down on a microscope slide well covered with 10 µl PAS. Amoebae were allowed to attach for 30 min, and the well was washed once with PAS, followed by fixation with 4% formaldehyde (12 min at room temperature). An endosymbiont-specific probe (PDD8-644, 5′-TCTTCGACTCCAGCCGCAC-3′) was designed using the ARB software package and validated with probeCheck and Silva testProbe 3.0^57, 58^. The probe sequence was deposited at probeBase (http://probebase.net/pb_report/probe/4042)^59^. All probes were synthesized by Thermo Fisher Scientific (St. Leon-Rot, Germany). Hybridizations were performed by combining the specific probe PDD8-644, the eukaryotic probe EUK516 (5′-ACCAGACTTGCCC TCC-3′)^60^, and the bacterial probe set EUB338 I-III (5′-GCTGCCTCCCGTAGGAGT-3′, 5′-GCAGCCACCCGTAGGTGT-3′ and 5′-GCTGCCACCCGTAGGTGT-3′)^60, 61^. Hybridization was carried out for 2 h at 46 °C with 20% formamide using standard hybridization and washing buffers^62^. Slides were embedded in Citifluor prior to examination with a confocal laser scanning microscope (LSM 510 Meta, Zeiss, Oberkochen, Germany) or an epifluorescence microscope (Axioplan 2 Imaging, Zeiss) equipped with a CCD camera (AxioCam HRc, Zeiss).

### DNA extraction, PCR, cloning and sequencing

Amplification and sequencing of the amoebal 18S rDNA was carried out as described earlier^37^. Briefly, trophozoites from clonal cultures were harvested from plates with cotton swabs and re-suspended in 15 ml centrifuge tubes filled with 5 ml 0.9% sodium chloride (NaCl). The samples were centrifuged for 10 min at 800 × g, the supernatants were discarded and the pellets were re-suspended in 200 µl 0.9% NaCl. Total genomic DNA was extracted from the cells using the QIAmp® DNA Mini Kit (Qiagen, Hilden, Germany). The entire 18S rDNA gene was amplified using the newly designed Cochlio-1 primer (5′-CCTGGTTGATCCTGCCAG-3′) and the SSU2 (5′-TCCTGATCCTTCTGCAGGTTCAC-3′)^63^. Gel bands were extracted with the GFX PCR DNA and Gel Band Purification Kit (GE Healthcare, UK) and overlapping fragments were directly sequenced in both directions with the ABI PRISM® BigDye sequencing kit, using the internal primers P1fw 5′-CAAGTCTGGTGCCAGCAGC-3′, P1rev 5′-GCTGCTGGCACCAGACTTG-3′, P2fw 5′-GATCAGATACCGTCGTAGTC-3′, P2rev 5′-GACTACGACGGTATCTGATC-3′, P3fw 5′-CAGGTCTGTGATGCCCTTAG-3′ and P3rev 5′-CTAAGGGCATCACAGACCTG-3′^64^ and an ABI PRISM 310® automated sequencer (PE Applied Biosystems, Germany). A consensus sequence was built with the GeneDoc sequence editor^65^.

In order to sequence the bacterial 16S rRNA gene, amoebae were collected from an *E. coli*-depleted culture flask, and 2 ml of the suspension were transferred to a 2 ml microcentrifuge tube. DNA extraction of the amoeba culture was carried out using the DNeasy Blood and Tissue Kit (Qiagen, Hilden, Germany). Amplification of bacterial 16S rRNA genes was performed using primers 616F (5′-AGAGTTTGATYMTGGCTCAG-3′) and 1492R (5′-GGYTACCTTGTTACGACTT-3′) at 52 °C annealing temperature and 35 cycles^66, 67^. PCR reactions contained 100 ng template DNA, 50 pmol/µl of each primer, 1 unit of Taq DNA polymerase (Fermentas, St. Leon-Rot, Germany), 10x Taq buffer with KCl and 2 mM MgCl_2_, and 0.2 mM of each deoxynucleotide in a total volume of 50 µl. PCR products were purified using the PCR Purification Kit (Qiagen) and subsequently cloned using the TOPO XL Cloning Kit (Invitrogen, Darmstadt, Germany) per manufacturer’s recommendations. Sanger sequencing of four clones was performed by Microsynth Austria.

### Phylogenetic analysis

The obtained 16S rRNA gene sequence was subjected to sequence homology search against the nr/nt database using the BLASTn service available at the NCBI website^68^. The top ten high scoring sequences with a minimum length of 1,400 nt were downloaded, and phylogenetic analysis was performed together with a selection of related taxa retrieved from the SILVA ribosomal RNA gene database^69^. The SINA aligner^70^ with standard settings and variability set to “Bacteria” was used for sequence alignment. The alignment was trimmed at both ends to only include positions covered in all sequences. Pairwise sequence similarity was calculated using ARB^57^. Phylogenetic trees were calculated using PhyloBayes^71^ with the CAT model^72^ and GTR exchange rates. Ten independent chains were calculated with 210 generations each. For the final converged tree, all 10 chains were taken into account whereas the first 20 generations trees were removed. iTOL v3^73^ was used to edit and label the tree.

### Data availability

DNA sequences determined in this study were deposited at Genbank/ENA/DDBJ under accession numbers KU215597 (18S rRNA gene sequence of *C. minus* 9B) and LT716083 (16S rRNA gene sequence of ‘*Candidatus* Cochliophilus cryoturris’ PDD8).
